# Whole-Genome Sequence of Salmonella enterica subsp. diarizonae Serovar 61:k:1,5,(7) Strain 1569 (14PM0011), Isolated from German Sheep

**DOI:** 10.1128/MRA.00539-19

**Published:** 2019-09-19

**Authors:** Anne Busch, Herbert Tomaso, Ulrich Methner

**Affiliations:** a Friedrich-Loeffler-Institut, Institute of Bacterial Infections and Zoonoses (IBIZ), Jena, Germany; University of Maryland School of Medicine

## Abstract

Here, we report the draft genome sequence of Salmonella enterica subsp. diarizonae serovar 61:k:1,5,(7) strain 1569, alternatively named 14PM0011, which is a common serovar in German sheep that is unrepresented in the databases and considered and described as being host adapted with low virulence.

## ANNOUNCEMENT

Salmonellosis is an infectious disease caused by bacteria of the genus Salmonella. The disease occurs in both animals and humans and represents one of the most important zoonoses. The species Salmonella enterica is divided into the following 6 subspecies: S. enterica subsp. enterica (I), S. enterica subsp. salamae (II), S. enterica subsp. arizonae (IIIa), S. enterica subsp. diarizonae (IIIb), S. enterica subsp. houtenae (IV), and S. enterica subsp. indica (VI) (1). Salmonella enterica subsp. diarizonae serovar 61:k:1,5,(7) belongs to subspecies IIIb. Although this serovar is considered host adapted, it displays a very different epidemiological pattern than does the sheep-restricted Salmonella enterica serovar Abortusovis. *Salmonella* serovar 61:k:1,5,(7) is able to produce both intestinal and extraintestinal infections with fecal, vaginal, and nasal colonization but mostly without clinical disease (2). These properties deviate from the classical characteristics of host-restricted, host-adapted, or ubiquitous serovars. Therefore, the term “sheep-associated serovar” (2) appears to be appropriate for characterizing *Salmonella* serovar 61:k:1,5,(7).

Here, we report the whole-genome sequence of Salmonella enterica subsp. diarizonae serovar 61:k:1,5,(7) strain 1569, isolated from a fecal sample from sheep in Thuringia, Germany, in 2014. Bacterial procedures and serotyping were performed as described before (2, 3). Antimicrobial susceptibility of the strain was assessed by determining the MIC by using the broth microdilution method with Sensititre EUVSEC plates (Trek Diagnostic Systems Ltd., East Grinstead, United Kingdom). Epidemiological cutoff values were used according to the European Committee on Antimicrobial Susceptibility Testing (EUCAST) (4).

The MIC value (μg/ml) of the strain for each antimicrobial was as follows: >512 (sulfamethoxazole), 1 (trimethoprim), 0.06 (ciprofloxacin), 4 to 8 (tetracycline), <0.03 (meropenem), 8 to 16 (azithromycin), 8 (nalidixic acid), <0.25 (cefotaxime), <8 (chloramphenicol), 1 to 2 (tigecycline), <0.05 (ceftazidime), <1 (colistin), 2 (ampicillin), and 1 (gentamicin). MIC values (μg/ml) for the antimicrobials indicate resistance as follows: ≥512 (sulfamethoxazole), ≥4 (trimethoprim), ≥0.12 (ciprofloxacin), ≥16 (tetracycline), ≥0.25 (meropenem), ≥64 (azithromycin), ≥32 (nalidixic acid), ≥1 (cefotaxime), ≥32 (chloramphenicol), ≥2 (tigecycline), ≥4 (ceftazidime), ≥4 (colistin), ≥64 (ampicillin), and ≥4 (gentamicin). ResFinder (5) predicted an acquired antimicrobial resistance to aminoglycoside.

Strains were grown overnight at 37°C in 5 ml of Luria-Bertani broth and adjusted to an optical density at 580 nm (OD_580_) of 0.3 by dilution with sterile Luria-Bertani broth. Genomic DNA was isolated using Qiagen Genomic-tip 20/G and a Qiagen genomic DNA buffer set kit (Hilden, Germany). DNA libraries were constructed using the Nextera XT DNA preparation kit, and paired-end sequencing was performed on the MiSeq platform (Illumina, Inc., San Diego, CA, USA) using a 600-cycle MiSeq reagent kit. Quality checking was performed with FastQC. In total, 990,500 reads were generated. Reads were *de novo* assembled using SPAdes 3.12.0 in Bayes Hammer mode (–careful) (6) and evaluated with QUAST v4.3 (7) with standard settings. Filtering of the sample was performed by removing contigs with coverage less than 5× and a size below 500 bases. The genome assembly was represented by 66 contigs with an *N*_50_ contig length of 158,533 bp, in which the largest contig had 372,036 bp. The average coverage was >90-fold. The combined length of the contigs was 4,794,677 bp with a G+C content of 51.36%. Annotation was performed with Prokka 1.12 (8). Annotation features include 4,448 DNA coding sequences (CDSs), 9 rRNAs, 76 tRNAs, and 1 transfer-messenger RNA (tmRNA). These data are in concordance with those of other reported Salmonella enterica isolates.

### Data availability.

The whole-genome sequence of Salmonella enterica subsp. *diarizonae* serovar 61:k:1,5,(7) strain 1569, also known as 14PM0011, was submitted to NCBI with the RefSeq assembly accession number GCF_005280635, to the SRA under accession number SRX5804671, and to GenBank under accession number SZWB00000000. The version described in this paper is the first version.
